# Parallel processing streams for motor output and sensory prediction during action preparation

**DOI:** 10.1152/jn.00616.2014

**Published:** 2014-12-24

**Authors:** Max-Philipp Stenner, Markus Bauer, Hans-Jochen Heinze, Patrick Haggard, Raymond J. Dolan

**Affiliations:** ^1^Wellcome Trust Centre for Neuroimaging, University College London, London, United Kingdom;; ^2^Department of Neurology, University of Magdeburg, Magdeburg, Germany;; ^3^Institute of Cognitive Neuroscience, University College London, London, United Kingdom; and; ^4^Max Planck UCL Centre for Computational Psychiatry and Ageing Research, London, United Kingdom

**Keywords:** sensory attenuation, sensory prediction, motor priming, lateralized readiness potential, agency

## Abstract

Sensory consequences of one's own actions are perceived as less intense than identical, externally generated stimuli. This is generally taken as evidence for sensory prediction of action consequences. Accordingly, recent theoretical models explain this attenuation by an anticipatory modulation of sensory processing prior to stimulus onset (Roussel et al. 2013) or even action execution (Brown et al. 2013). Experimentally, prestimulus changes that occur in anticipation of self-generated sensations are difficult to disentangle from more general effects of stimulus expectation, attention and task load (performing an action). Here, we show that an established manipulation of subjective agency over a stimulus leads to a predictive modulation in sensory cortex that is independent of these factors. We recorded magnetoencephalography while subjects performed a simple action with either hand and judged the loudness of a tone caused by the action. Effector selection was manipulated by subliminal motor priming. Compatible priming is known to enhance a subjective experience of agency over a consequent stimulus (Chambon and Haggard 2012). In line with this effect on subjective agency, we found stronger sensory attenuation when the action that caused the tone was compatibly primed. This perceptual effect was reflected in a transient phase-locked signal in auditory cortex before stimulus onset and motor execution. Interestingly, this sensory signal emerged at a time when the hemispheric lateralization of motor signals in M1 indicated ongoing effector selection. Our findings confirm theoretical predictions of a sensory modulation prior to self-generated sensations and support the idea that a sensory prediction is generated in parallel to motor output (Walsh and Haggard 2010), before an efference copy becomes available.

the brain is thought to predict the sensory consequences of one's actions (Wolpert and Flanagan 2001). One phenomenon generally taken as evidence for sensory prediction of action consequences is sensory attenuation (Bays et al. 2006; Wolpert and Flanagan 2001). Sensory attenuation refers to a decrease in perceived intensity of stimuli that are self-generated. This decrease is often explained by a cancellation of predicted reafferent sensory signals (e.g., Cullen 2004; but see Brown et al. 2013). Attenuation phenomena have been demonstrated in the human somatosensory (Chapman et al. 1987), visual (Cardoso-Leite et al. 2010) and auditory (Weiss et al. 2011b) systems, as well as in other sensory modalities across species (for a review, see Cullen 2004). While attenuation phenomena in humans have been localized to sensory cortices (Blakemore et al. 1998; Martikainen et al. 2005), the question how sensory cortex function differs when a stimulus is self-generated remains controversial (see, e.g., Brown et al. 2013). According to recent theoretical models, sensory attenuation is explained by an anticipatory modulation of sensory processing that occurs before the expected stimulus (Roussel et al. 2013) or even before execution of the action that causes the stimulus (Brown et al. 2013). More generally, these models raise the interesting question at which point a modulation of sensory cortex function occurs during an action. Sensory prediction is often assumed to depend on an efference copy of the motor command (Wolpert and Flanagan 2001). This implies that a sensory modulation should occur only after motor output has been specified.

While some previous studies have argued for efference-based prediction (e.g., Blakemore et al. 1999; Gentsch and Schütz-Bosbach 2011; Weiss et al. 2011b), typical study designs often include some potential confounds. For example, in a typical design, perception of stimuli that are caused by one's own actions is compared to perception of identical stimuli that are consequences of someone else's actions (e.g., Weiss et al. 2011a, 2011b) or generated by a machine (e.g., Shergill et al. 2003). In many of these studies, it is difficult to control stimulus predictability, task-relevance and task load (performing an action or not). Not controlling for these factors can complicate the interpretation of attenuation phenomena (for a review, see Hughes et al. 2012). Importantly, these factors may also influence prestimulus sensory signals (Alink et al. 2010; Bauer et al. 2012; Jensen et al. 2002; Rohenkohl and Nobre 2011; Tuladhar et al. 2007). These designs cannot easily isolate prestimulus effects specifically related to causing a stimulus.

To avoid these potential confounds, we used an implicit manipulation of the sense of agency that allowed us to hold stimulus predictability, task-relevance and task load (motor output) constant. “Sense of agency” refers to a subjective experience that one's actions control events in the outside world (Haggard and Chambon 2012). A stronger subjective experience of agency is associated with stronger sensory attenuation (Desantis et al. 2012). Here, to vary sensory attenuation, we used an established manipulation of the sense of agency based on subliminal motor priming. Motor priming influences action selection: a subliminal prime stimulus, presented shortly before a suprathreshold target, introduces a transient action selection bias (Dehaene et al. 1998; Eimer and Schlaghecken 1998, 2003). Priming effects critically depend on the delay between primes and targets. Specifically, at short stimulus onset asynchronies (SOA), a prime that instructs the same response as a subsequent target (compatible priming) results in motor performance benefits (Vorberg et al. 2003). This effect reverses at longer SOA, i.e., compatible primes lead to motor performance costs [negative compatibility effect (NCE)], thought to result from auto-inhibition of the initially primed response (Eimer and Schlaghecken 2003). Importantly, beyond these effects on motor performance, priming is increasingly recognized as a useful tool to study the subjective experience of an action and its consequence. In particular, recent studies show that compatible priming enhances subjective agency ratings, both at short and long prime-target SOA (Chambon and Haggard 2012; Wenke et al. 2010). In line with this, we have previously shown psychophysically that compatible NCE-priming of an action enhances sensory attenuation of its consequence (Stenner et al. 2014).

In the present study, motor priming allowed us to obtain a de-confounded perceptual metric of sensory attenuation. Motor priming specifically targets the processes of action selection and preparation, while the action that is eventually executed and its sensory consequence can be held constant. As a result, changes in stimulus predictability, task-relevance and motor task load (performing an action) can be avoided. By combining magnetoencephalography (MEG) and psychophysics in a priming paradigm, we tested for a modulation of sensory cortex signals prior to a stimulus whose perception is attenuated, as predicted by recent theoretical models (Brown et al. 2013; Roussel et al. 2013).

NCE-priming also provides a chronometric marker of ongoing motor command specification, reflected in the lateralization of motor cortical signals (Eimer and Schlaghecken 1998, 2003). Specifically, primes first produce an initial activation of motor cortex contralateral to the primed response (Fig. 1*B*, *top*, “I”). If the prime-target SOA is sufficiently long, this is rapidly followed by a reversal (Fig. 1*B*, *top*, “II”) and activation of ipsilateral motor cortex (Eimer and Schlaghecken 2003). This second priming stage gives rise to the NCE on motor performance: if the instructed response and the primed response are the same, motor lateralization must reverse again before the correct action can be executed. The time course of these lateralized motor signals, therefore, informs about the currently prepared action.

**Fig. 1.**
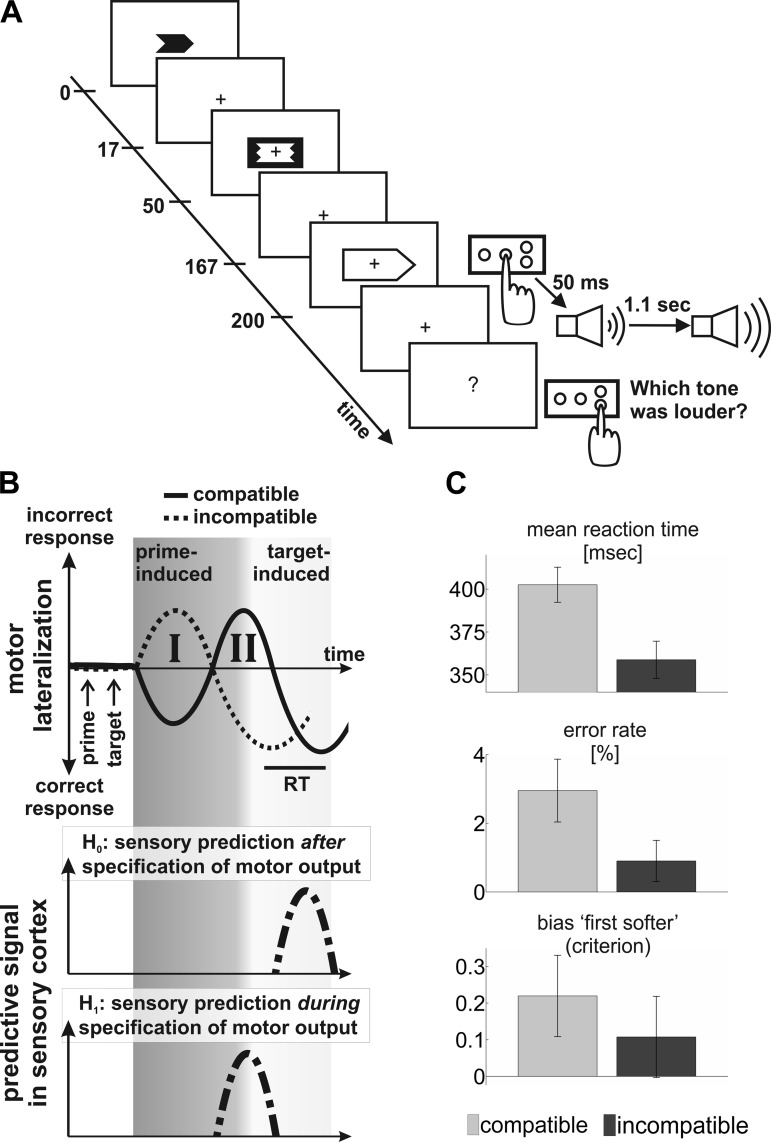
Schematic of one trial, competing hypotheses and behavioral results. *A*: schematic of one trial (compatible condition). Numbers on the timeline represent time in milliseconds after the onset of the prime arrow. *B*, *top*: schematic time courses of lateralized motor signals during negative priming (Eimer and Schlaghecken 1998). Dotted line, incompatible condition; solid line, compatible condition. For both conditions, time courses are plotted so that lateralization at the time of the correct response to the target [black bar, reaction time (RT)] falls below the *x*-axis. Dark gray shading: motor lateralization is dominated by the prime. I, initial activation of motor cortex contralateral to the primed response; II, subsequent activation of ipsilateral motor cortex (Eimer and Schlaghecken 2003). Light gray shading: motor lateralization is influenced by the target. Note that motor lateralization lags behind the physical onset of primes and targets (black arrows). *Middle*: according to models of efference-based prediction, a sensory prediction becomes available only after effector (hand) selection is complete (light gray shading). *Bottom*: a sensory prediction becomes available while effector (hand) selection is ongoing (dark gray shading). *C*: mean RTs (*top*, in ms), error rates (*middle*, in %) and bias to report the first tone as the softer (*bottom*, criterion) across compatible (light gray) and incompatible (dark gray) trials. Error bars represent the SE of the mean. Compatible and incompatible conditions differ significantly in all three measures (see results).

We asked how the latency of any putative prestimulus signal in sensory cortex might depend on this process of motor command specification. In principle, this signal could follow (or coincide with) the final reversal of lateralized motor cortical signals (Fig. 1*B*, *middle*). This would be compatible with the idea that a sensory prediction alters sensory cortex function after motor output has been fully specified, as implied by models of efference-based prediction (Wolpert and Ghahramani 2000). Alternatively, a prestimulus sensory signal could precede this reversal of motor lateralization (Fig. 1*B*, *bottom*). This would support the idea of parallel processing streams for motor output and sensory prediction, as proposed before (Walsh and Haggard 2010).

## METHODS

### Participants

Seventeen right-handed, healthy volunteers participated for payment (mean age = 25.4 yr; SD = 5.4; nine women). Two participants were excluded due to conscious perception of the primes (see *Behavioral Data Analysis*). All subjects gave written, informed consent prior to participation. Participants were naive as to the purpose of the experiment. The study was approved by the local ethics committee (University College London, UK).

### Task and Stimuli

The paradigm (Fig. 1*A*) was similar to a task in a recent study (Stenner et al. 2014). The aim was to vary the degree of sensory attenuation of an auditory action consequence by subliminal priming of the action. Subjects performed an action which caused a tone and then had to judge the loudness of this tone. The crucial experimental manipulation was subliminal priming of the hand used to perform the action.

The experiment consisted of four parts: a training session, an adaptive staircase procedure, the main experiment and a control task, in this order. Details of the adaptive staircase procedure and the control task are described in the footnotes. During the main experiment and the training session, participants made a speeded response to a visual target arrow on a screen by pressing a button with their left or right index finger, as instructed by the direction of the target (Fig. 1*A*). Each target arrow was preceded by a subliminal prime arrow, which pointed in the same or in the opposite direction as the target (compatible and incompatible conditions, respectively; each in 50% of all trials). Due to metacontrast backward masking, the prime was not consciously perceived, as confirmed by a forced-choice recognition test at the end of the experiment. The SOA between the prime and the target was 200 ms. This SOA is well known to result in a NCE, i.e., in motor performance costs when the prime and target point in the same direction (Lingnau and Vorberg 2005).

In trials in which participants pressed the wrong button in response to the target or in which they pressed too late (>1.2 s after target onset), a red “x” was presented, and the trial ended without presentation of a tone. These trials were repeated at the end of a block of 32 trials. Trials with an incorrect or no response to the target were excluded from all analyses (except to determine the corresponding error rate).

A correct button press triggered the presentation of two successive tones after 50 ms and 1,150 ms, respectively. The onset times of both tones were therefore equally predictable. Each tone was delivered binaurally for 100 ms via air tubes to in-ear headphones. Each of the two buttons participants could press in response to the target was associated with a different tone pitch (900 Hz vs. 750 Hz). On any given trial, the pitch of both tones was determined by the button participants pressed (e.g., for a given participant, a left button press always resulted in a high-pitch tone and a right button press always resulted in a low-pitch tone). The reason for using distinct tone pitches for the two buttons was to enhance a sense of agency for the consequences of pressing either button and to motivate tone prediction on the basis of effector selection. The mapping between buttons and tone pitches was counterbalanced across subjects. Each participant learned this mapping in a training session of 32 trials before the main experiment. To ensure that participants attended to the mapping throughout the training session, between three and five catch trials were introduced in which the mapping was reversed. Participants had to count these catch trials (note that catch trials and the associated task of counting them were only present in the training session, not in any of the other parts of the experiment).

We obtained a perceptual metric of sensory attenuation by asking participants to compare the loudness of the two tones at the end of each trial and to indicate which of the two was perceived as louder (the first or second). Sensory attenuation is limited to a short time window of a few hundred milliseconds around the onset of an action (Bays et al. 2005). Because of this dependence on temporal contiguity, we predicted that sensory attenuation would affect perception of the first tone more strongly than perception of the second, introducing a bias to report the first tone as the softer (see *Bias as a Measure of Sensory Attenuation* in the discussion). In line with previous findings (Stenner et al. 2014), this bias was expected to be stronger in compatible than incompatible trials.

Sound pressure level (SPL) was fixed at 74-dB SPL for the first tone and varied across trials for the second. Two SPLs were used for the second tone, either louder or softer than 74-dB SPL. These levels were determined for each subject using an adaptive staircase procedure before the main experiment (Kaernbach 1991).^1^ Subjects indicated which of the two tones was louder by pressing one of two additional buttons with the right middle finger. To avoid any direct interference between responses to the visual target and responses in the loudness discrimination task, these additional buttons were arranged orthogonal to the ones pressed in response to the target. Response time in the loudness discrimination task was not limited.

During the main experiment compatible and incompatible trials were pseudorandomly interleaved and equally frequent. Participants completed 12 blocks of the task (∼40 min in total). At the end of each block (32 trials), the average percentage of correct responses in the loudness discrimination task was displayed on the screen to motivate performance. In total, each participant completed 192 trials of the main experiment in each condition (excluding trials in which subjects pressed the wrong button in response to the target arrow or did not respond to the target within 1.2 s).

#### Stimuli.

All stimuli were generated and presented using Presentation software (Neurobehavioral Systems, www.neurobs.com). Visual stimuli were presented on a white background. Each trial started with the presentation of a prime arrow for 17 ms (solid black arrow, 2.84° × 1.18° visual angle, pointing to the left or right). The prime was followed by a metacontrast backward mask after 33 ms (interstimulus interval), which stayed on the screen for 117 ms and consisted of a black rectangle framing two white superimposed arrows pointing in both directions (3.18° × 1.66° visual angle). Thirty-three milliseconds after mask offset, a target arrow was presented for 117 ms. Targets were black arrow outlines pointing to the right or to the left (5.3° × 1.95° visual angle). A fixation cross (font size 1°) remained on the screen throughout the entire experiment (except for the response interval for the discrimination task, for which it was replaced by a question mark). All visual stimuli were presented at fixation [targets were slightly shifted horizontally in the direction in which they pointed (by 0.675°), so that the “trunk” of the arrow (excluding the tip) was centered]. Visual stimuli were projected onto a screen in the MEG recording room (vertical refresh rate of 60 Hz), with a viewing distance between participant and screen of ∼75 cm. The delay between programmed and physical onset of visual stimuli was found to be one frame (∼17 ms) and constant with millisecond precision as revealed by prior photodiode testing.

Sound volume was calibrated to decibels SPL using a SPL meter. Playback latency of the sound card was determined to be below 1 ms.

### Behavioral Data Analysis

Data on loudness discrimination in the main task and on prime recognition in the control task^2^ were analyzed using signal detection theory (Green and Swets 1966). Effects of prime-target compatibility on reaction time (RT), error rates and bias (criterion) were tested with one-tailed, dependent samples *t*-tests across subjects. One-tailed tests were used in these three analyses because the direction of each of the predicted effects was previously established (Eimer and Schlaghecken 1998; Stenner et al. 2014).

Results from previous studies suggest that priming effects on both sensory attenuation (Stenner et al. 2014) and the sense of agency (Damen et al. 2014; Wenke et al. 2010) depend on prime perception being unconscious. Our aim was therefore to exclude participants who perceived the primes consciously, at least to some extent. To this end, we tested each subject's performance in the prime recognition control task against chance-level performance using a nonparametric resampling test. For each trial, a response (“left prime,” “right prime”) was drawn randomly and with replacement from all responses of that subject in the prime recognition task. d′ was then recomputed for the resampled data. This was repeated 10^5^ times for each subject to obtain a nonparametric distribution under the null hypothesis of chance-level performance. In two subjects, actual d′ values in the prime recognition task exceeded the 95% confidence interval of this null distribution [d′ of 0.85, *P* = 0.0009 (male) and 0.76, and *P* = 0.0019 (female)]. Both participants also reported seeing the primes in at least some of the trials. These participants were therefore excluded from analysis (see *Unconscious or Conscious Prime Processing?* in the discussion). All reported results are qualitatively unchanged when including all 17 participants.

### MEG Recording and Analysis

MEG data were recorded continuously from 274 axial gradiometers and 35 reference channels of a CTF Omega system at a sampling frequency of 600 Hz. Head position was measured continuously via three coils at the nasion and the preauricular points. MEG was only recorded during the main experiment, not during the training, the staircase or the prime recognition test. Recording was paused every 10–12 min for a short break of about 1 min. Participants completed a total of three of these 10- to 12-min sessions.

#### Epoching and artifact rejection.

Data analysis used FieldTrip (Oostenveld et al. 2011) and SPM12b (Litvak et al. 2011). MEG data were epoched into trials that started 500 ms before prime onset and ended 200 ms after the response in the loudness discrimination task. Data were inspected visually using standard routines in FieldTrip (“ft_rejectvisual.m”) based on a threshold for amplitude variance for each trial. Remaining artifacts arising from eye movements were removed using principal component analysis. Artifact rejection was done blind to condition. After artifact rejection, 177 trials (range 150 to 193) were, on average, available for MEG analysis for each subject and condition. Line noise was removed from 6-s periods around each trial using a narrowband notch filter (48.5 to 51.5 Hz; 4th-order, two-pass Butterworth filter).

#### Realignment and planar gradients at the sensor level.

For sensor-level analysis, artifact-free data were interpolated to a common sensor array template across subjects to correct for interindividual variations in head positions (“ft_megrealign.m”). To better estimate the cortical topography of differences in the MEG field between conditions at the sensor-level, a planar gradient representation of the *t*-statistic was calculated (Bastiaansen and Knösche 2000). To this end, a nearest-neighbor interpolation method was used in FieldTrip, based on the first-order spatial derivative of the signal (“ft_megplanar.m”, followed by “ft_combineplanar.m”). Note that planar gradients were used for visualization purposes only, not in statistical analyses.

#### Source reconstruction.

Source reconstruction was based on individual T1-weighted MRI warped to the standard Montreal Neurological Institute (MNI) brain in SPM12b, using an inverse spatial normalization to fit a canonical MNI mesh with 8,196 dipoles to the position of each individual's cortical sheet (“spm_eeg_inv_mesh.m”). This forward modeling approach has the benefit that reconstructed activity can be assigned to homologous sources across subjects, as described in detail before (Litvak and Friston 2008). For each individual participant, MEG data were first merged across sessions (“spm_eeg_merge.m”, which computes an average of sensor and fiducial positions across sessions). Merged MEG data and individually fitted MNI meshes were then co-registered based on the positions of the fiducials (“spm_eeg_inv_datareg_ui.m”). Lead fields were computed on the basis of a single-shell volume conduction model (“spm_eeg_inv_forward.m”; Nolte 2003). We used a minimum-norm estimate (i.e., an identity matrix as the prior source covariance) to project the sensor data onto the cortical grid in SPM12b (“spm_eeg_invert.m”).

#### Statistical analysis.

Statistical analysis of the amplitude of MEG fields was based on a nonparametric randomization test (Maris and Oostenveld 2007) implemented in FieldTrip, which controls for type I errors by correcting for multiple comparisons across sensors and time bins. Nonparametric randomization tests have been widely used in previous studies of MEG data (e.g., Maris and Oostenveld 2007; Wang et al. 2012). First, clusters comprising adjacent sensors and time bins which exceeded a threshold of the *t*-statistic for a given contrast were defined (*P* < 0.05, two-tailed dependent samples *t*-test across subjects). A cluster-level statistic was derived for each cluster in the observed data by summing *t*-values across its elements (separately for clusters with positive and negative *t*-values). The null hypothesis was rejected if this cluster-level statistic exceeded a critical value, which was determined by the distribution of the maximum cluster-level statistic after repeatedly permuting the observed data (500 permutations), i.e., after randomly reassigning the data to the two conditions within subjects and determining the cluster-level statistic. *P* values were defined as the proportion of randomizations for which the maximum cluster-level statistic exceeded the cluster-level statistic in the observed data. *P* values were considered significant if they exceeded a two-tailed threshold of *P* < 0.05. Note that this method clusters adjacent time bins on the basis of a first-level statistic, so that the temporal cluster extent may vary slightly for identical contrasts based on slightly different data (e.g., between the sensor- and source-level).

For visualization of statistical differences at the source-level, an *F*-contrast was used instead of a *t*-contrast. The reason for this was that dipole orientation was normalized to the cortical surface [in alignment with the orientation of pyramidal cells in the cortex, the assumed dominant generators of the MEG signal (Okada et al. 1997)]. This leads to opposite orientations and, therefore, opposite signs of *t*-values for dipoles on adjacent surfaces of the same gyrus.

#### Time interval of interest and auditory localizer topography.

We predicted a modulation of auditory cortex signals by motor priming during action selection, i.e., prior to motor execution and to auditory stimulus presentation. To test this prediction, we time-locked MEG epochs to the onset of the prime arrow. We focused on effects of prime/target compatibility on time-locked signals between 300 ms after prime onset (i.e., 100 ms after target onset) and tone onset (on average across conditions 630 ms after the prime). Note that the initial stage of prime-related motor lateralization (“I” in Fig. 1*B*, *top*, and see Fig. 4*A*, *bottom*) peaked at ∼150 ms after target onset (see Fig. 4*A*, *bottom*), i.e., behavioral adaptation to visual cues lagged behind visual stimulation.

We studied the topography of these effects in relation to an auditory localizer. As a localizer signal, we used the MEG signal 50 to 150 ms after onset of the second tone, relative to the average signal across a 50-ms baseline interval before the second tone. Note that this interval is 1,200 to 1,300 ms after the button press in the priming task, at a time when motor signals related to the button press were unlikely to interfere with the auditory localizer. To formally test the topographical similarity of the priming effect to the auditory localizer at the sensory level, we first calculated, for each subject, the correlation coefficient between (axial gradiometer) topographies across all sensors. These correlation coefficients were then Fisher *z*-transformed and tested against zero in a one-sample *t*-test across subjects.

All results of the nonparametric randomization tests are corrected for multiple comparisons across all time bins between 300 ms after prime onset and onset of the tone. In addition, all sensor-level results are corrected for multiple comparisons across all 274 sensors. The high spatial resolution of our source reconstruction allowed us to test for effects of compatibility in a region of interest (ROI) in auditory cortex, where we expected a predictive sensory signal to emerge. This ROI was defined based on the auditory localizer, i.e., by a contrast that was independent of the effect of compatibility. For this ROI, we sorted grid points within 25 mm of the peak of the auditory localizer according to their *F*-value in the localizer contrast. For each hemisphere, we chose the largest contiguous cluster among the 150 grid points with the highest localizer *F*-values. The MNI coordinates of the localizer peaks were [−55 −45 11] (left hemisphere) and [56 −31 11] (right hemisphere). The MEG signal was averaged across each ROI separately after taking into account opposite dipole orientations on adjacent surfaces of the same gyrus. To this end, the signal at each cortical grid point in the ROI was multiplied by the sign of the auditory localizer *t*-statistic at that grid point. This increases spatial specificity by enhancing signals with a similar spatial orientation as the auditory localizer.

#### Motor lateralization.

Previous EEG studies have characterized time-dependent changes in the lateralized readiness potential (LRP) during subliminal motor priming (Eimer and Schlaghecken 1998), which are widely held to mark distinct stages of action selection (Eimer and Schlaghecken 2003). Here, prime-induced lateralization of motor cortical MEG signals provided a chronometric marker of ongoing motor command (hand) specification. To obtain a time course of motor lateralization in MEG, we used an approach that is similar to a well-established method for computing the LRP from EEG data, called the double-subtraction method (Eimer 1998). For EEG data, average signals from two electrodes, overlying right and left central regions (usually C4' and C3'), are subtracted in a first step to obtain lateralized time-series. This is done separately for left- and right-hand movements. In a second step, the difference in (signed) lateralization between left- and right-hand movements is calculated. The resulting time course corresponds to the LRP. For MEG data, a similar subtraction of average signals across trials with a left- vs. right-hand movement has been used before to obtain a time course of motor lateralization for each hemisphere (Praamstra et al. 1999). In the case of simple finger movements, like the ones studied here, these lateralized MEG signals have been localized to primary motor cortex, specifically to the motor hand area (Praamstra et al. 1999). Here, we subtracted, for each condition, the average signal across trials with a right-hand movement from the average signal across trials with a left-hand movement at two cortical grid points in mirror-symmetric locations in the left and right motor hand area, identified as the hand “knob” on the precentral gyrus (hand “omega”; Yousry et al. 1997). These grid points were located at *x*, *y*, *z* = [−32 −19 61] (left) and [34 −17 59] (right) (MNI coordinates). Note that virtually identical time courses of motor lateralization were obtained across different pairs of mirror-symmetric grid points on the hand “knob,” confirming the validity of this estimate. We obtained a single time course of motor lateralization for each priming condition by subtracting the resulting right- and left-hemispheric signals, similarly to the subtraction of the average EEG signal at electrodes C3 and C4 in the computation of the LRP (Eimer 1998). The time courses of the resulting MEG signal in compatible and incompatible trials closely resembled the LRP in EEG studies of subliminal motor priming (Eimer and Schlaghecken 1998).

#### Matching of RT.

To exclude the possibility that differences in the amplitude of time-locked MEG signals between compatible and incompatible trials reflect unspecific differences in RTs rather than direct effects of the experimental prime-target compatibility manipulation, we reanalyzed data after matching trials across conditions for RT. To this end, we first calculated the matrix of absolute differences in RT between each trial in the compatible condition and each trial in the incompatible condition, separately for each participant. Next, we drew the pair of (compatible and incompatible) trials with the smallest absolute difference in RT without replacement. This was repeated until the number of matched pairs equaled the number of trials in the condition that had fewer trials (note that the number of trials which were included in MEG analyses varied slightly between the two conditions due to artifact rejection). We selected all trial pairs whose RT differences were smaller than a prespecified cut-off value, which was chosen to retain a maximum number of trials while eliminating any significant differences in RT. Independent-samples *t*-tests within subjects showed that RT-matching was successful: while 16 of the 17 participants showed a significant slowing of RT in compatible vs. incompatible trials before RT-matching, this effect was abolished in all participants after matching. On average across subjects, each condition consisted of 122 trials (range 91 to 151) after RT-matching.

## RESULTS

### Behavioral Results

We were first interested in effects of motor priming on the perceived loudness of the tone immediately caused by the action. In line with previous findings (Stenner et al. 2014), we predicted stronger attenuation when this action was instructed by a target that was compatible with the preceding prime. Since sensory attenuation is limited to a short time window of a few hundred milliseconds around the onset of an action (Bays et al. 2005), stronger sensory attenuation of the first tone would result in a stronger bias to report the first tone as the softer.

As expected, we replicated a NCE on motor performance (Eimer and Schlaghecken 1998). When primes and targets were compatible, mean RT in response to the target arrow was significantly slower [*t*(14) = 8.8, *P* < 0.001, one-tailed; Fig. 1*C*, *top*], and participants pressed the incorrect button significantly more often [*t*(14) = 2.17, *P* = 0.024, one-tailed; Fig. 1*C*, *middle*]. Crucially, we also observed a motor priming effect on bias in the loudness discrimination task (criterion). Specifically, the bias to report the first tone as the softer was significantly larger following compatible vs. incompatible priming of the action [*t*(14) = 2.24, *P* = 0.021, one-tailed; Fig. 1*C*, *bottom*]. As expected (see discussion), there was no significant effect of motor priming on discriminability of the loudness of the two tones as measured by d′ [*t*(14) = 0.86, *P* = 0.2, one-tailed; mean ± SE: 2.28 ± 0.09 (compatible) and 2.21 ± 0.11 (incompatible)].

### MEG Results

Using MEG, we examined whether the degree of sensory attenuation of the first tone is reflected in a modulation of neuronal activity in auditory cortex before tone onset, as predicted (Brown et al. 2013; Roussel et al. 2013). Our analysis proceeded in four steps. First, to relate any prestimulus effect of motor priming at the sensor-level to auditory processing, we compared its topography to the topography of an auditory localizer. Second, to examine whether, as predicted, priming modulates the prestimulus signal in auditory cortex, we performed a source-level ROI analysis. Third, to ensure that priming effects on auditory cortex are not explained by differences in RT across priming conditions, we compared compatible and incompatible conditions after matching trials for RT. And finally, to relate any compatibility effect in auditory cortex to ongoing motor command specification, we compared its time course to time-dependent changes in the lateralization of motor signals prior to motor execution.

### Motor Priming Effect on the MEG Signal in Auditory Cortex

**Fig. 2.**
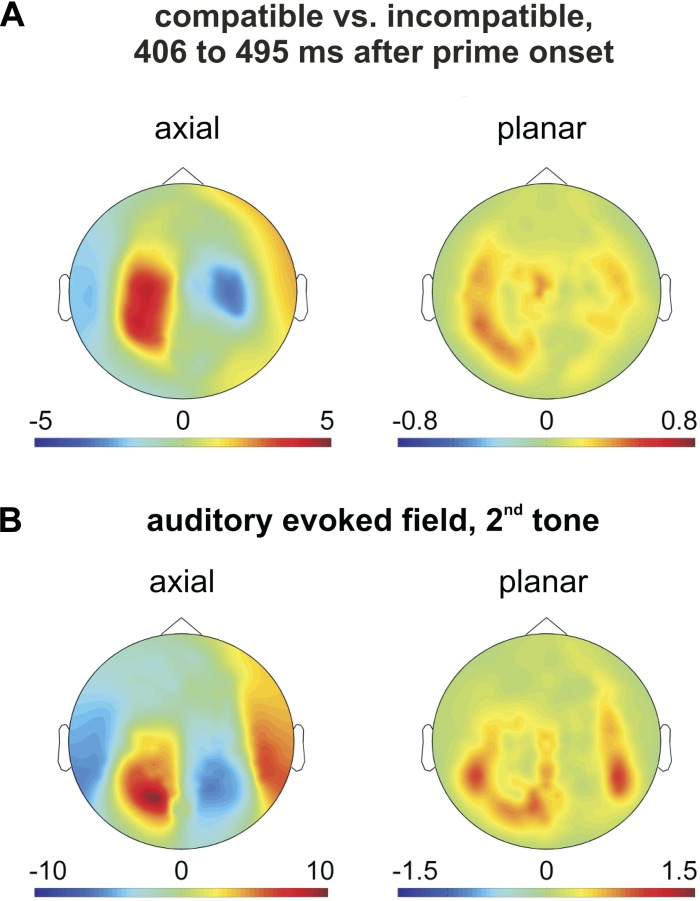
Sensor-level topographies of the compatibility effect and of the auditory localizer. Color codes *t*-values (*A*, *left*, and *B*, *left*) and arbitrary units (*A*, *right*, and *B*, *right*). *A*: sensor-level topography for axial gradiometers (*left*) and reconstructed planar gradiometers (*right*) of the effect of prime-target compatibility on the prime-locked magnetoencephalography (MEG) signal between 406 and 495 ms after prime onset. *B*: sensor-level topography for axial gradiometers (*left*) and reconstructed planar gradiometers (*right*) of the auditory localizer (50 to 150 ms after onset of the second tone, relative to a 50-ms prestimulus interval).

At the sensor-level, we found a cluster of left temporo-parietal and central sensors with significantly larger amplitude of the evoked field in compatible vs. incompatible trials between 406 and 495 ms after prime onset (*P* = 0.004; two-tailed, dependent-samples *t*-test across subjects, corrected for multiple comparisons across all sensors and all time bins between 300 ms after prime onset and onset of the tone). Note that, on average, participants did not press the button to trigger the first tone until 603 ms (compatible) and 559 ms (incompatible) after prime onset. Figure 2*A* shows the sensor-level topography of this effect for axial gradiometers (*left*) and reconstructed planar gradiometers (*right*). Note that the planar gradiometer representation used here permits a better estimation of the spatial distribution of underlying cortical generators, as it represents local cortical activity underneath corresponding sensors (Bastiaansen and Knösche 2000).

We compared the sensor-level topography of this effect of prime compatibility to the sensor-level topography of an auditory localizer. As a localizer, we used the MEG signal between 50 and 150 ms after onset of the second tone. Figure 2*B* shows the axial (*left*) and planar (*right*) gradiometer representation of this auditory localizer. Note the topographical overlap, particularly in the left hemisphere, between the planar representations of the localizer (2*B*, *right*) and of the compatibility effect (2*A*, *right*). To formally test this overlap, we calculated, for each subject, the Fisher *z*-transformed correlation coefficient between the (axial gradiometer) topographies of the compatibility effect and of the auditory localizer (across all sensors). A one-sample *t*-test across subjects showed that correlation coefficients were significantly greater than zero [*t*(14) = 2.95, *P* = 0.01, two-tailed].

**Fig. 3.**
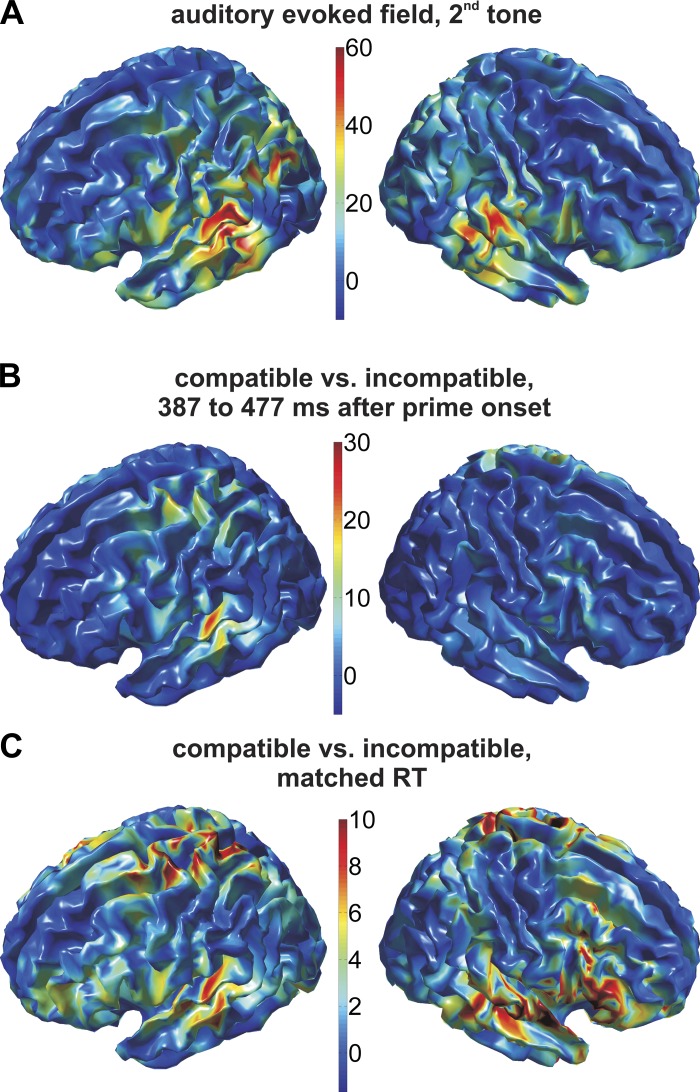
Source-level topographies of the compatibility effect and of the auditory localizer. Color codes *F*-values. *Left* column: lateral view on the left hemisphere; *right* column: lateral view on the right hemisphere. *A*: source-level topography of the auditory localizer, with the same time window as in Fig. 2*B*. *B*: source-level topography of the prime-target compatibility effect between 387 and 477 ms after prime onset. *C*: source-level topography of the prime-target compatibility effect, after matching trials for RT across priming conditions, between 413 and 463 ms after prime onset. Differences between time windows in *B* and *C* and Fig. 2*A* reflect slight variations in the temporal extent of clusters identified by cluster-based permutation testing, i.e., are purely data-driven (see methods). The topography of the prestimulus prime-target compatibility effect (*B*) overlaps with the auditory localizer (*A*) in left auditory cortex, irrespective of any differences in RT across conditions (*C*).

To examine this topographical overlap with greater spatial resolution, we performed source-reconstruction of the MEG data using a minimum-norm estimate. Figure 3*A* shows the cortical distribution of the auditory localizer in the same time window as used for the sensor-level analysis. As expected, the auditory localizer activated left and right auditory cortex. Importantly, we found that priming significantly modulated the signal in left auditory cortex between 387 and 477 ms after prime onset, with a higher amplitude of the prime-locked signal in compatible vs. incompatible trials (Fig. 3*B*; *P* < 0.001; dependent-samples *t*-test across subjects, corrected for multiple comparisons across all time bins between 300 ms after prime onset and onset of the tone). There was no significant modulation by compatibility in right auditory cortex (*P* = 0.24). Across the whole brain, the highest *F*-value was observed in left auditory cortex {at [−50 29 8] (MNI coordinates), Brodmann area 41, primary auditory cortex}. There was a second, weaker focal source around left sensorimotor cortex (peak *F*-value at [−23 −18 68], Brodmann area 6).

### Motor Priming Effects on Auditory Cortex Are Independent of RT

Importantly, we observed a similar pattern of results as in Fig. 3*B* when matching compatible and incompatible trials for RT (Fig. 3*C*; see methods). After matching, mean RTs across subjects were 383 vs. 379 ms for the compatible and incompatible condition, respectively, compared with 403 ms vs. 357 ms before RT-matching. Despite this, we found virtually the same spatial cluster of grid points in left auditory cortex with a higher amplitude of the time-locked signal in compatible vs. incompatible trials. After correcting for multiple comparisons across all time bins between 300 ms after prime onset and onset of the tone, there was a marginally significant cluster of time bins between 413 and 463 ms after prime onset (*P* = 0.052). Note that, by matching for RT, we retained only a subset of trials, which reduced the signal-to-noise ratio and may account for the fact that the cluster was only marginally significant after correction. Note also that the topographical pattern of the compatibility effect was hardly changed when matching trials for RT, in particular in the left hemisphere (compare Figs. 3, *B* and *C*, *left*). In summary, cortical sources of the prestimulus compatibility effect and of the auditory localizer overlapped in left auditory cortex, irrespective of any differences in RT between conditions.

### Temporal Relation of Auditory and Motor Signals

**Fig. 4.**
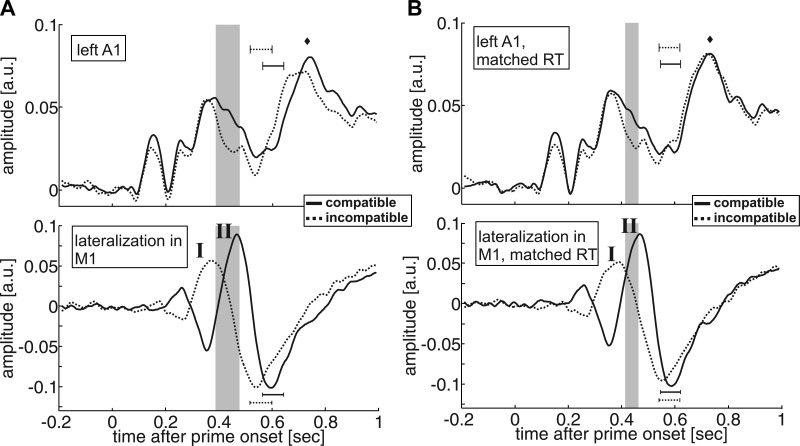
Time courses of auditory cortex signals and lateralized motor signals. In all plots, the shaded area represents the time window during which the amplitude of the signal in left auditory cortex is significantly higher in compatible vs. incompatible trials (the cluster extent in time in the nonparametric randomization tests, see results; the shading in *A* and *B* corresponds to the same time windows as in Fig. 3, *B* and *C*, respectively). The solid and dotted horizontal bars represent the mean (±SD) RT in the compatible and incompatible condition, respectively. The diamond in *A* and *B*, *top*, represents the mean expected latency of the auditory M100 (∼100 ms after stimulus onset). Solid line, compatible trials; dotted line, incompatible trials. All time courses are smoothed with a square kernel of 20 ms for visualization. *A*, *top*: time courses of prime-locked signals in left auditory cortex; *bottom*, lateralized motor cortical signals [pressed left minus pressed right, left- vs. right-hemispheric motor hand area; c.f., lateralized readiness potential (Praamstra et al. 1999)]. In the results, we describe motor lateralization with respect to two successive time windows, marked here by I and II. *B*: same as *A*, but after matching compatible and incompatible trials for RT. The prime-target compatibility effect (*A*, *top* and *B*, *top*) emerges at a time when the specification of the motor command is ongoing, as indexed by a dominant influence of the prime on motor lateralization (*A*, *bottom* and *B*, *bottom*, I and II.). a.u., Arbitrary units.

Next, we examined the temporal dynamics of this compatibility effect on neuronal activity in auditory cortex in relation to those of lateralized signals in the motor system during action selection. Figure 4, *A* and *B*, *top*, shows the time course of the signal in left auditory cortex for compatible and incompatible trials before (*A*) and after (*B*) matching trials for RT across priming conditions. Lateralized motor signals were computed separately for the compatible and incompatible condition (see methods). Note that the same response was preceded by primes that pointed in opposite directions in compatible vs. incompatible trials. Consequently, motor signals in the two conditions were expected to lateralize in opposite directions in response to the primes, but converge at the time of motor execution (Eimer and Schlaghecken 2003). Figure 4*A*, *bottom*, shows the time courses of these lateralized motor cortical signals (Fig. 4*B*, *bottom*, shows the same after matching trials for RT across priming conditions). These time courses strongly resembled the LRP in previous EEG studies of negative motor priming (Eimer and Schlaghecken 1998). Specifically, we replicated the two phases of motor lateralization described in the Introduction (marked by “I” and “II” in Fig. 4, *A* and *B*, *bottom*, corresponding to “I” and “II” in Fig. 1*B*, *top*). We describe motor lateralization during these phases in relation to motor lateralization at the time of motor execution (horizontal bars in Fig. 4, *A* and *B*, *bottom*). In the first phase, primes induced motor lateralization in favor of a response that was eventually executed in compatible trials and rejected in incompatible trials. During the second phase, motor lateralization reversed in favor of a response that was subsequently rejected in compatible trials and confirmed in incompatible trials. This second, prime-induced motor bias is thought to account for motor performance benefits in incompatible trials and costs in compatible trials at long prime target SOA (Eimer and Schlaghecken 2003).

Interestingly, the compatibility effect in auditory cortex (Fig. 4*A*) emerged at the very start of this second phase of prime-induced motor lateralization. This indicates that the modulation of neuronal activity in auditory cortex emerged while motor lateralization was still dominated by the prime, and not yet by the target, suggesting that the specification of motor output was still ongoing at this point.

## DISCUSSION

Our study demonstrates that neuronal activity in sensory cortex is modulated in anticipation of a sensory action consequence, as predicted by theoretical accounts of sensory attenuation (Brown et al. 2013; Roussel et al. 2013). We show that this modulation, together with a perceptual metric of sensory attenuation, varies with an established manipulation of the sense of agency (Chambon and Haggard 2012; Wenke et al. 2010). Furthermore, the latency of this sensory modulation relative to preparatory motor cortical signals supports the idea of parallel processing streams for sensory prediction and motor output.

### Motor Priming and Sensory Attenuation

Sensory attenuation is generally taken as evidence for predictive action control (Bays et al. 2006; Wolpert and Flanagan 2001). However, interpreting attenuation phenomena is often complicated by potential confounds. In particular, studies which compare self-generated and externally generated stimuli are likely to introduce additional differences in stimulus predictability and motor task load (for a review, see Hughes et al. 2012). Our priming paradigm avoided these likely confounds. On every trial, subjects performed an action that had a predictable auditory consequence. Since a manipulation of action preparation varied the degree of sensory attenuation, our perceptual metric of sensory attenuation likely reflects a predictive process that is intimately related to motor actions (Stenner et al. 2014).

Previous work has shown that subliminal motor priming influences a subjective experience of control over a stimulus (Chambon and Haggard 2012; Wenke et al. 2010). More specifically, compatible priming is known to enhance the subjective experience of agency. In agreement with the idea that sensory attenuation is influenced by a subjective belief of agency (Desantis et al. 2012), we find that compatible priming also results in stronger sensory attenuation.

### Parallel Processing of Sensory Prediction and Motor Output

The idea that the brain predicts sensory consequences of one's own actions has a long tradition (Helmholtz 1886; Holst and Mittelstaedt 1950; Sperry 1950). In line with this tradition, contemporary computational models emphasize the importance of sensory prediction for motor control (Adams et al. 2013; Wolpert and Ghahramani 2000). According to a pervasive theory in the field, sensory prediction is based on an efference copy of the motor command (Franklin and Wolpert 2011; Wolpert and Ghahramani 2000). In effect, this theory makes a strong assumption regarding the earliest stage during motor preparation at which a sensory prediction can alter sensory cortex function, namely after a motor command has been specified.

To date, findings relating to the hierarchical level of motor processing at which a sensory prediction is generated have been inconsistent. While several studies in humans (Haggard and Whitford 2004; Voss et al. 2008, 2009; Walsh and Haggard 2010) and monkeys (Seki and Fetz 2012) suggest that sensory attenuation (as an indicator of sensory prediction) is influenced by motor processing upstream of primary motor cortex, others emphasize the role of M1 (Therrien et al. 2011; Voss et al. 2007).

Here, we report neurophysiological evidence for a partial dissociation of sensory prediction from motor output. We demonstrate an anticipatory signal in sensory cortex prior to motor execution. The timing of this sensory signal relative to lateralized motor cortical signals does not comply with the idea that a motor command must be fully specified before a sensory prediction can alter sensory cortex function, as assumed in efference copy models. Instead, it suggests that sensory prediction and motor output are organized in parallel processing streams, similar to action specification and selection in a recent parallel processing framework (Cisek and Kalaska 2010). Two previous studies arrived at a similar conclusion by combining a sensory attenuation paradigm with a Go/NoGo task and a stop-signal task (Walsh and Haggard 2007, 2010). Walsh and Haggard (2007) observed recovery from sensory attenuation over the course of 200 ms after the presentation of a NoGo signal when participants successfully stopped a speeded reaction. On unsuccessful stop trials, they found a second phase in which sensory attenuation was renewed prior to the error of executing an action (2010). Importantly, the authors found the first phase of temporary, partial recovery from sensory attenuation even in stop trials in which erroneous movements eventually occurred. These findings suggest dissociable processing streams for sensory prediction and motor output. Furthermore, flexibility seems to be higher in the processing stream that predicts sensory consequences, both in adapting to an unexpected change in task requirements (the stop-signal) and in integrating the final state of motor output (upon errors of commission). In accordance with this higher flexibility, our own study demonstrates that an anticipatory modulation in sensory cortex integrates a motor-relevant cue, the target arrow, before it influences imminent motor output.

### Sensory Attenuation in Other Modalities

In our study, the causal association between an action and its sensory consequence was based on an abstract rule that was mediated by a computer and learned before the task. In contrast, proprioceptive, mechanoreceptive and, in the case of head movements, vestibular sensations are inherent to motor output. Previous studies have found no sensory attenuation when self-applied force or self-initiated head motion are controlled via a joystick or a steering wheel, i.e., according to abstract rules (Roy and Cullen 2001; Shergill et al. 2003). This suggests that predictability of somatosensory or vestibular sensations based on motor output is in itself insufficient to produce sensory attenuation (conversely, we show that sensory attenuation can vary even when stimulus predictability on the basis of motor output is constant). Furthermore, these findings imply that attenuation of somatosensory and vestibular sensations depends on an immediate, “natural” way of causing these sensations, e.g., during self-touch or via contraction of neck muscles, respectively. We note, however, that at least one previous study reported attenuation of a somatosensory stimulus even though the action-outcome association in this case was indirect (Tsakiris and Haggard 2003).

Auditory action consequences, on the other hand, often result from an interaction with the world, i.e., they often reflect a context-dependent, learned contingency. In contrast to somatosensory or vestibular reafference, auditory action consequences are attenuated even when the underlying action-outcome contingency follows an abstract rule (Weiss et al. 2011a, 2011b). An interesting explanation for this discrepancy between sensory modalities could be that somatosensory and vestibular signals are more directly relevant for motor control, specifically via reflex arcs. When somatosensory or vestibular action consequences are mediated by a machine (e.g., when a monkey rotates his body in space by steering a mechanical turntable, as in Roy and Cullen 2001), postural adaptation to unexpected contingency changes (e.g., when the speed of the turntable changes unexpectedly) benefits from a preserved gain of proprioceptive and vestibular reflexes. In contrast, when proprioceptive or vestibular sensations are inherent to movement, e.g., during active head-on-body movements, contingencies are stable and only depend on the integrity of the body and the functioning of sensory systems. Under these circumstances, somatosensory and vestibular sensations can, therefore, be attenuated.

This heterogeneity of sensory attenuation phenomena across modalities is often neglected in the previous literature. Models that were developed to explain sensory attenuation of somatosensory reafference, in particular efference copy models (e.g., Blakemore et al. 1999), are often also cited to explain sensory attenuation in other sensory modalities (e.g., Weiss et al. 2011b). However, the question whether efference-based prediction mechanisms generalize across modalities has not been directly tested before. We identified an anticipatory signal in auditory cortex at a time when the specification of motor output is ongoing. This finding questions the notion of a general, amodal efference-based prediction mechanism.

### Bias as a Measure of Sensory Attenuation

We used bias in a discrimination task as a measure of sensory attenuation. Many previous studies interpret sensory attenuation as an underestimation bias that is independent of a change of sensory gain, using similar discrimination tasks (Desantis et al. 2012; Haggard and Whitford 2004; Stenner et al. 2014; Tsakiris and Haggard 2003; Weiss et al. 2011b). Our interpretation of bias as a sensory, rather than purely decisional, parameter is in agreement with recent evidence that bias (criterion) can in fact reflect the baseline activity of signal-selective units at an early, sensory stage, in addition to, or instead of, decisional processes at late response stages (Wyart et al. 2012). Absence of a priming effect on sensitivity (discriminability) in our study, on the other hand, makes a potential, confounding effect of priming on attention unlikely. Attention would be expected to affect the sensory gain function (Hillyard et al. 1998) and, consequently, discriminability (discrimination sensitivity, d′).

### Unconscious or Conscious Prime Processing?

While we found no statistically significant recognition of prime direction in the 15 participants who were included in our study, this does not prove entirely unconscious processing of primes in these subjects. Indeed, criteria used to determine whether conscious stimulus awareness is entirely absent or not are a matter of ongoing debate (e.g., Erdelyi 2004; Hannula et al. 2005). However, the focus of our study was not to address the question whether effects of motor priming on sensory attenuation depend on entirely unconscious or merely low levels of prime awareness. Instead, we utilized motor priming to investigate the influence of motor preparation on sensory prediction and sensory attenuation while avoiding potential confounds like stimulus predictability. In view of previous studies which suggest that clearly visible primes vs. masked primes can have effects in opposite directions (Damen et al. 2014; Stenner et al. 2014; Wenke et al. 2010), we excluded subjects who performed significantly above chance in the prime recognition task, following standard procedures in the priming literature (e.g., Sumner et al. 2007). All reported results are unchanged when including all 17 participants.

### Laterality of the Auditory Cortex Signal

We find an anticipatory signal in left, but not right, auditory cortex. The laterality of this finding may, in principle, reflect a true functional lateralization or a false negative result for right auditory areas. There is evidence for a left-hemispheric dominance in motor preparation (Kim et al. 1993; Rushworth et al. 2001), including stronger effects of priming on the excitability of left motor cortex (Verleger et al. 2006). Our finding that priming modulates the signal in the motor hand area [the hand “omega” (Yousry et al. 1997)] exclusively in the left hemisphere confirms this left-hemispheric dominance. In addition, there is evidence for a stronger interconnection of auditory and motor functions in the left than the right hemisphere (Aziz-zadeh et al. 2004). In line with this, previous studies in humans (Sanmiguel et al. 2013) and monkeys (Brosch et al. 2005) have reported signals in left auditory cortex in anticipation of an auditory action consequence, which are compatible with our own finding.

### Conclusions

In summary, our results confirm a theoretical prediction that neuronal activity in sensory cortex is modulated in anticipation of a sensory action consequence. Furthermore, our study supports the idea of two parallel, partially dissociable processing streams during action preparation: one for motor output and one for sensory prediction.

## GRANTS

This work was supported by the Wellcome Trust (Ray Dolan Senior Investigator Award 098362/Z/12/Z). M. P. Stenner was supported by a scholarship from the German Research Foundation (Deutsche Forschungsgemeinschaft DFG, STE 2091/1-1). P. Haggard was supported by an Economic and Social Research Council Professorial Fellowship, and by European Research Council Advanced Grant HUMVOL (Human Volition, Agency and Responsibility). The Wellcome Trust Centre for Neuroimaging is supported by core funding from the Wellcome Trust 091593/Z/10/Z.

## DISCLOSURES

No conflicts of interest, financial or otherwise, are declared by the author(s).

## AUTHOR CONTRIBUTIONS

Author contributions: M.-P.S. and M.B. conception and design of research; M.-P.S. performed experiments; M.-P.S. analyzed data; M.-P.S., M.B., and P.H. interpreted results of experiments; M.-P.S. prepared figures; M.-P.S. drafted manuscript; M.-P.S., M.B., P.H., and R.J.D. edited and revised manuscript; M.-P.S., M.B., H.-J.H., P.H., and R.J.D. approved final version of manuscript.

## Footnotes

1 Before the main experiment, SPL differences between the first and the second tone were individually adjusted using a weighted up/down method (3 up/1 down) (Kaernbach 1991). Subjects performed virtually the same task as in the main experiment, with a few differences, as described in the following. On any given trial, the SPL either of the first or second tone was 74-dB SPL, while the other tone (the comparison tone) was louder or softer than 74-dB SPL. The SPL of the comparison tone was adapted exponentially, approaching 74-dB SPL asymptotically from above and below with improving performance. At any point of the staircase, the SPL of the comparison tone was determined by (74 dB SPL ± 6 dB SPL × 1.15^exponent^). Exponents started at 0 (corresponding to differences in SPL between the first and the second tone of ±6-dB SPL). Exponents were decreased by 1 after a correct response and increased by 3 after an incorrect response. There were four independent, interleaved staircases, which approached 74-dB SPL from above or below, separately for the two tone pitches (900 and 750 Hz). In total, the staircase procedure consisted of 6 blocks of 32 trials each. During the staircase procedure, participants received visual feedback after each loudness discrimination judgment (a green tick or a red “x” at fixation). Note that this feedback was only given during the staircase, not during the main experiment. At the end of the staircase procedure, discrimination thresholds were determined by averaging all exponents at reversal points, for each of the four staircases separately. Throughout the staircase procedure, primes were neutral (pointing in both directions).

2 After the experimental task, existence of the primes was revealed to participants, and a forced choice prime recognition test was added as a control task. Visual stimulation was identical to the experimental task, but participants were instructed to ignore the direction of the target arrows, as well as the mask, and to focus on the direction of the primes only. They were required to indicate whether primes pointed to the right or the left on each trial (by using the respective index finger buttons). There was no time limit for these responses. Instead, participants had to wait for a tone (875 Hz, duration 150 ms, presented 600 ms after the onset of the target) before responding. This constraint for response times was introduced to keep the influence of subliminal priming on the button choice to a minimum and follows procedures used in previous studies for the same purpose (Chambon and Haggard 2012; Vorberg et al. 2003; Wenke et al. 2010). There was no feedback for this prime recognition task. The prime recognition task consisted of 3 blocks, each of 32 trials.
